# 3D-engineered WO_3_ microspheres assembled by 2D nanosheets with superior sodium storage capacity†

**DOI:** 10.1039/d4ra01800a

**Published:** 2024-05-14

**Authors:** Shilpi Sengupta, C. Sudakar, Manab Kundu

**Affiliations:** a Electrochemical Energy Storage Laboratory, Department of Chemistry, SRM Institute of Science and Technology Chennai 603203 Tamil Nadu India manab.kundu@inl.int; b Multifunctional Materials Laboratory, Department of Physics, Indian Institute of Technology Madras Chennai 600036 India

## Abstract

Because of the inadequate sodium storage capacity of graphite, the exploration of high-performance SIB anodes is a crucial step forward. Herein, we report the hydrothermally synthesized self-assembled interconnected nanosheets of WO_3_ microspheres possessing admirable sodium storage in terms of cycling stability and acceptable rate capability. Benefitting from the interconnected nature of the nanosheets with a hollow interior, the WO_3_ microspheres exhibited a high sodiation capacity of 431 mA h g^−1^ at 100 mA g^−1^ and an excellent rate performance of 60 mA h g^−1^ at 500 mA g^−1^ with an impressive coulombic efficiency of around 99%. Importantly, even after continuous cycling with increasing current densities, a specific capacity as high as 220 mA h g^−1^ could be recovered at a current density of 50 mA g^−1^, suggesting excellent sodium storage reversibility.

## Abstract 2

This work focuses on the self-assembled interconnected nanosheets of WO_3_ microsphere anode for sodium-ion batteries, delivering high capacity and excellent rate performance due to their interconnected structure and hollow interior.

## Graphical abstract

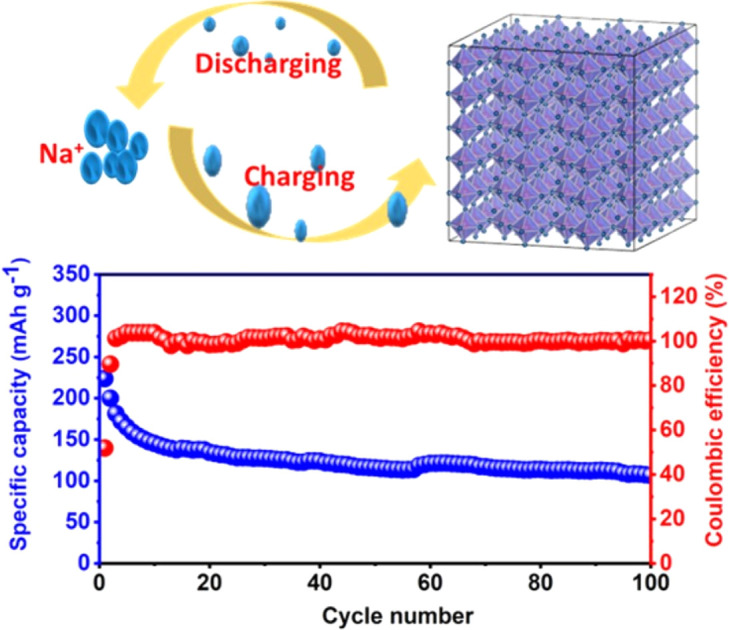

## 1. Introduction

Concerns about the depletion of fossil-based resources worldwide can only be effectively addressed with the help of renewable, clean energy technologies.^1–4^ Energy storage technologies play an important role in the success of renewable energy integration.^5,6^ Lithium-ion batteries (LIBs), which have been used to power portable electronics for decades, are currently considered the best option for powering the next generation of electric vehicles and stationary applications. However, the limited reserves (0.006 wt% abundance on the earth) and the rising cost of lithium present significant obstacles to the widespread commercial production of LIBs.^7^ In contrast, sodium (the 4th most abundant element with 2.64 wt% abundance on the earth) resources are abundant and accessible everywhere in the world. For large-scale and stationary-storage applications, SIBs with similar electrochemical storage mechanisms have emerged as potential alternative energy storage systems.^8^ Similar to LIBs, the final performance (energy and power density) of SIBs will be dependent on their electrode materials. Therefore, a step forward for SIBs would be the development of new anode materials with higher capacities and better cycling stability.^5,9^ In recent years, the scientific community has shifted its focus to other materials, particularly conversion-type metal-ion batteries with transition metal oxide electrodes, where the different oxidation states of a compound may offer a high specific capacity for advanced applications.^10,11^ Cobalt oxides, nickel oxides, manganese oxides, ruthenium oxides, and tungsten oxides have been explored extensively owing to the variable oxidation states of metal ions, which facilitate redox reactions and charge storage.^12–16^ WO_3_ has recently been investigated as an anode material because of its electrochemical stability, diversity of crystalline phases, non-toxic nature, and high conductivity. From a fundamental standpoint, WO_3_ could be an ideal model system to study intercalation-initiated conversion reactions.^17,18^ Moreover, the morphology of the electrode is another key parameter in enhancing the capacity of the material by shortening the ion diffusion length. Various research groups have explored tungsten oxide at the nanometer scale with different morphologies, including nanowires,^18^ nanorods,^19^ and nanosized irregular particles.^20^ Santhosha *et al.* synthesized GO–WO_3_*via* the sol–gel method, and for the first time, reported a sodiation capacity of 927 mA h g^−1^ at 30 mA g^−1^.^21^ Fanyan Zeng *et al.* synthesized WO_3−*x*_ nanorods with *in situ*-hybridized oxygen vacancies in nitrogen-doped carbon nanosheets, which delivered specific capacities of 230.8 mA h g^−1^ and 184.6 mA h g^−1^ at 20 A g^−1^ and 30 A g^−1^, respectively.^22^ Because of only limited reports with WO_3_, the sodium storage mechanism and the effects of microstructures are not yet clearly understood.

In this work, we have synthesized hierarchical 3D WO_3_ microspheres composed of very thin nanosheets using a facile hydrothermal method without any surfactant. The average thickness of the WO_3_ nanosheets is ∼5 nm. The staggered arrangement of interconnected ultrathin nanosheets forming hollow microspheres can provide more active sites to ease the electrolyte infiltration and enhance the contact between the active materials and electrolytes. Since microspheres have larger surface areas, they can provide more sites for Na^+^ storage, reduce the effects of volume changes, make Na^+^ intercalation into active materials easier, and decrease the diffusion length. While assessing sodium storage behaviour, the as-prepared WO_3_ exhibited a high sodiation capacity of 431 mA h g^−1^ at 100 mA g^−1^, and excellent rate performance of around 60 mA h g^−1^ at even 500 mA g^−1^ with an impressive coulombic efficiency of around 99%.

### 1.1 Experimental section

Phase pure WO_3_ has been synthesized by a facile process using the hydrothermal method. Tungsten hexachloride (0.3 M WCl_6_) and thioacetamide (0.6 M C_2_H_5_NS) were dissolved in 40 ml of deionized water and stirred for around 1 h to get a clear solution. The resultant mixture was transferred into a 50 ml Teflon-lined stainless-steel autoclave and maintained in a preheated oven (180 °C) for 48 h. After naturally cooling to room temperature, the sample was collected, centrifuged, and washed, followed by annealing at 700 °C for 1 hour at a ramp rate of 5°.

The balanced chemical equation is as follows:2WCl_6_ + 9C_2_H_5_N_S_ → 2WO_3_ + 9CS_2_ + 12HCl

This equation indicates that 2 moles of WCl_6_ react with 9 moles of C_2_H_5_NS to produce 2 moles of WO_3_, 9 moles of CS_2_, and 12 moles of HCl.

The process commences with the nucleation of minute WO_3_ nanoparticles (Fig. S1†). This nucleation process initiates sheet formation, wherein individual atoms or molecules congregate to create small clusters of WO_3_ nanosheets. Subsequently, microspheres are generated through Ostwald ripening, a phenomenon elucidating the growth of larger particles at the expense of smaller ones within a solution. This growth mechanism arises from the differing solubilities between smaller and larger particles. In the context of tungsten trioxide, larger microspheres are augmented by assimilating material from adjacent smaller ones, resulting in a reduction in the number of smaller particles and a concurrent enlargement of larger ones over time.

Continuing through Ostwald ripening, some microspheres may amalgamate and coalesce, giving rise to larger structures. Under specific conditions, these structures organize into thin, sheet-like formations. The determinants of temperature, concentration, and solvent properties exert a pivotal influence on shaping the final morphology of tungsten trioxide.

### 1.2 Characterization

The morphological studies of the sample were performed using field-emission scanning electron microscopy (FESEM, Thermo Scientific Apreo S). The phase and crystal structures were analyzed using the X-ray diffraction pattern obtained using a diffractometer (XRD, BRUKER USA D8 Advance, Davinci). The Raman spectra were obtained using a HORIBA (France) LABRAM HR Evolution instrument and measurements were done using a laser source with an excitation wavelength of 514 nm. The surface chemical states were analyzed by X-ray photoelectron spectroscopy (XPS, physical electronics). High-resolution transmission electron microscopy (HR-TEM, JEOL Japan, JEM-2100 Plus) studies were also performed to discern the morphology and crystal structure at the nanoscale using high-resolution and the selected area electron diffraction pattern (SAED).

### 1.3 Electrochemical characterization

Cyclic voltammetry (CV) and galvanostatic charge–discharge (GCD) measurements were performed using a biologic electrochemical workstation in the potential range of 0.01–3.0 V. The same workstation was also used to do the electrochemical impedance spectroscopic (EIS) measurements. CR 2032-coin cells were assembled using glass fiber separators. Sodium was used as a counter electrode and 1 mol L^−1^ NaClO_4_ in ethylene carbonate (EC), ethyl methyl carbonate (EMC), and dimethyl carbonate (DMC) (1 : 1 : 1 by volume) was used as the electrolyte. The electrodes were fabricated using a slurry-coating method with the SP carbon and polyvinylidene fluoride (PVDF) in *N*-methyl-2-pyrrolidinone solvent in the weight ratio of 7.5 : 1.5 : 1.

## 2. Results and discussion

**Fig. 1:**
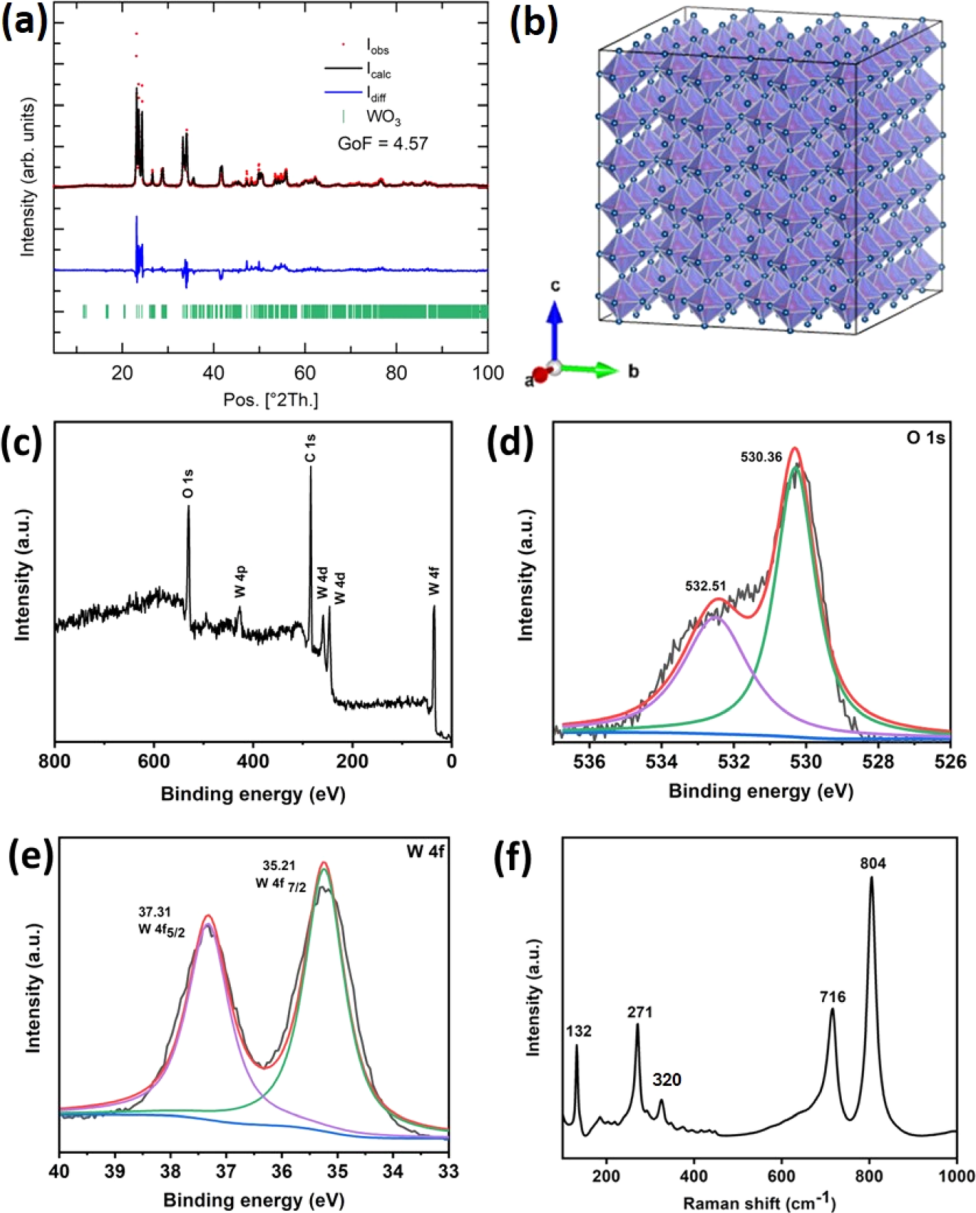
(a) A standard refinement plot illustrating the X-ray diffraction pattern of WO_3_. (b) A polyhedral representation of monoclinic WO_3_. (c) XPS survey spectrum of tungsten oxide (WO_3_). High-resolution XPS spectra of (d) oxygen (O 1s) and (e) tungsten (W 4f). (f) Raman spectrum of as-synthesized WO_3_.

**Fig. 2:**
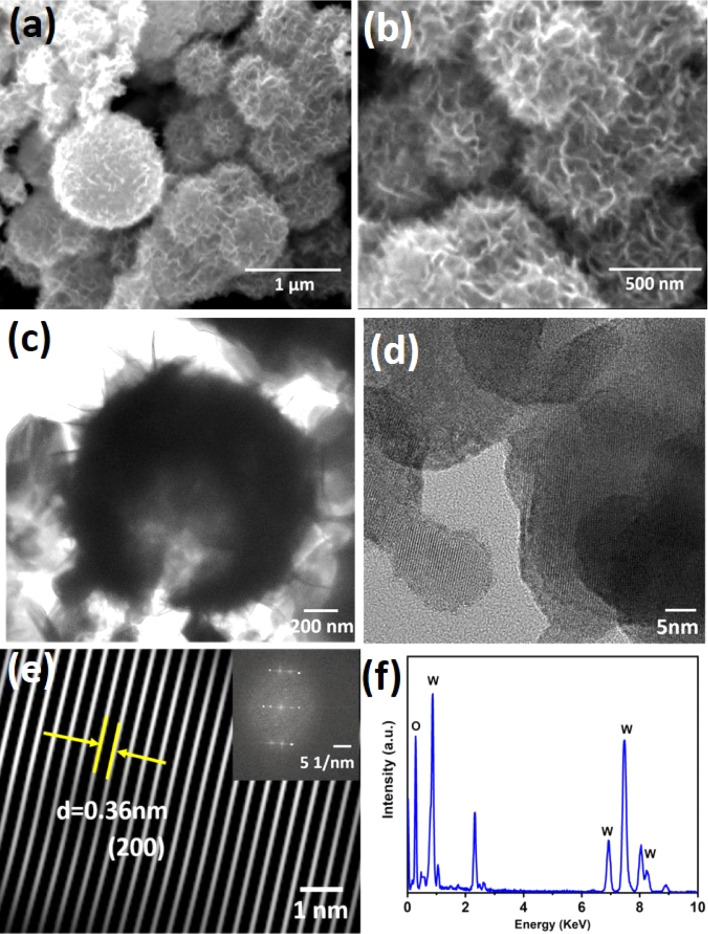
FESEM image of WO_3_ (a) at low magnification and (b) at high magnification. (c) Bright-field (d) high-resolution TEM images of WO_3_. (e) A filtered inverse FFT image showing the lattice plane. The inset shows the FFT (fast Fourier transform) pattern. (f) EDX (energy dispersive X-ray analysis) of the corresponding HRTEM image.

Fig. 1(a) depicts a standard Rietveld refinement plot for the X-ray diffraction pattern of the monoclinic phase of the WO_3_ sample. The experimental diffraction pattern is represented by symbols, while the refined one is depicted by a continuous line. The clear sharpness observed in the diffraction peaks not only indicates the high-quality crystallization of the synthesized WO_3_ but also the absence of any impurity peaks in the pattern. All the peaks in this spectrum can be perfectly indexed to a monoclinic phase of WO_3_. Fig. 2(b) depicts the crystal structure of monoclinic WO_3_ in a 3D view. The estimated lattice constants of *a* = 7.3008 Å, *b* = 7.5389 Å, and *c* = 7.6896 Å (JCPDS card # 75-2187) from the diffraction pattern match well with the reported structure of WO_3_.^23^

Using XPS, the chemical composition and oxidation states of the elements were determined. The XPS survey scan spectrum (Fig. 1(c)) shows the presence of W, O, and C. The deconvoluted O 1s core-level spectra are shown in Fig. 1(d). The W 4f (Fig. 1(e)) spectrum is proficiently divided into the tungsten orbitals W 4f_5/2_ and W 4f_7/2_, which have binding energies of 37.31 eV and 35.21 eV, respectively, confirming the presence of the W^6+^ oxidation state of WO_3_.^24,25^ The split between the W 4f_7/2_ and W 4f_5/2_ core levels is 2.1 eV, indicating a +6 oxidation state of W in WO_3_. The Raman spectrum of the as-synthesized WO_3_ structure is depicted in Fig. 1(f), showing the four major characteristic peaks in the 100 cm^−1^ to 900 cm^−1^ region. Two strong characteristic bands at 804 and 716 cm^−1^ represent the W–O–W stretching vibrations of the bridging oxygen from the monoclinic crystalline phase of WO_3_. The bands at 320 cm^−1^, 271 cm^−1^ and 132 cm^−1^ represent the bending vibrations of the (O–W–O) mode.^26,27^

The FESEM images (Fig. 2(a) and (b)) reveal that each microsphere consists of many interconnected nanosheets, which form a spherulitic microspherical hierarchical structure with a diameter ranging between 1.0 and 1.5 μm. The sheet-like internal microstructure remarkably increases the surface area, thus enhancing ionic diffusion. The nanosheets show hyperbranched flower structures due to subsequent nucleation. The interconnected nanosheets provide sufficient ionic channels for the electrolyte to establish large accessible internal surfaces for an electrochemical redox reaction, resulting in a high specific capacity.^28^

The very thin and transparent nanosheets were observed at the surface of the micro-sphere by transmission electron microscopy (TEM, Fig. 2(c)). The hollow nature of the microspheres was visible through a fracture that may have occurred during sample preparation by ultrasonication. Fig. 2(d) and (e) presents high-resolution TEM (HRTEM) displaying a lattice spacing of 0.36 nm corresponding to the (200) plane of WO_3_, which is in line with the XRD. The EDX spectra (Fig. 2(f)) also confirmed the presence of tungsten and oxygen in the sample.

N_2_ adsorption–desorption measurements were utilized to assess the specific surface areas and pore structures of WO_3_, as depicted in Fig. S2(a and b).† According to the IUPAC classification, the N_2_ adsorption–desorption isotherms exhibited pseudo-type IV hysteresis. The BET specific surface area of WO_3_ was determined to be 12.240 m^2^ g^−1^. Fig. S2(b)† illustrates pore-size distribution plots obtained using the BJH method for pristine WO_3_. The average BJH pore volume of pristine WO_3_ was measured at 0.045 cm^3^ g^−1^, indicating a broad pore-size distribution ranging from 19 nm to 30 nm, suggestive of the presence of macropores. The prepared WO_3_ microspheres demonstrated a high specific surface area and a favorable pore-size distribution.

**Fig. 3:**
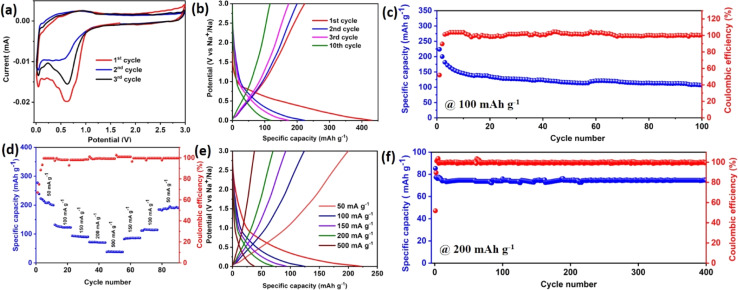
(a) Cyclic voltammogram (CV) at a sweep rate of 0.2 mV s^−1^. (b) Galvanostatic charge–discharge profiles of different cycles at a current density of 100 mA g^−1^. (c) Long cycling and coulombic efficiency at a current density of 100 mA g^−1^. (d) Rate performance and coulombic efficiency of WO_3_. (e) The corresponding charge–discharge profiles at different current densities are shown. (f) Long-term cycling at a current density of 200 mA h g^−1^ for up to 400 cycles.

The electrochemical studies were conducted *via* CV and GCD in a potential window of 0.01 V to 3 V (Fig. 3). The cyclic voltammogram for the first three consecutive cycles at a scan rate of 0.2 mV s^−1^ is presented in Fig. 3(a). The broad peak from ∼0.86 V to 0.29 V in the first cycle during the reduction process corresponds to the insertion of Na^+^ into the interlayers of WO_3_, followed by the peak at 0.62 V associated with the conversion of WO_3_ into elemental W and the irreversible SEI layer formation, as well as the insertion of Na^+^ (sodium ion) to form metallic W and Na_2_O. During the oxidation process, a broad peak was observed at ∼0.91 V due to the extraction of Na^+^ as well as the formation of W to WO_3_. From the 2^nd^ cycle onwards, the reduction and oxidation peaks appeared at 0.8 V and 0.7 V, respectively, denoting the conversion reaction between WO and W.

1 WO_3_ + 6Na^+^ + 6e^−^ ↔ 3Na_2_O + W

The charge and discharge profile for a few specific cycles at 100 mA g^−1^ is shown in Fig. 3(b). The first discharge and charge capacities were 431.3 mA h g^−1^ and 222.31 mA h g^−1^, respectively. A huge capacity deprivation with an initial coulombic efficiency of 51.8% was observed, due to the formation of a solid electrolyte interphase (SEI) on the surface of the electrode by the decomposition of the electrolyte.^29^ The theoretical capacities of carbon materials were less than 250 mA h g^−1^.^30^ The as-synthesized WO_3_ showed a better experimental capacity as compared to carbon materials. After the 10^th^ cycle, the capacity fading was stable up to the 100^th^ cycle. The coulombic efficiency was also enhanced with cycling and stabilized, showing ∼100%.

Besides the satisfactory specific capacity and superior cycling stability, the WO_3_ electrode also showed satisfactory rate capability. As displayed in Fig. 3(c and d), on increasing current densities from 50 to 100, 150, 200, and 500 mA g^−1^, reversible capacities of 222.5, 124.0, 92.8, 70.0 and 40 mA h g^−1^ were achieved, respectively. Interestingly, even after prolonged cycling at increasing current densities, a specific capacity of up to 220 mA h g^−1^ was recovered at a current density of 50 mA g^−1^, indicating outstanding sodium storage reversibility. A long cycling test was performed at a current density of 200 mA h g^−1^ for up to 400 cycles as shown in Fig. 3(e). The test showed a negligible capacity loss. The capacity observed after 400 cycles was ∼75 mA h g^−1^ with a coulombic efficiency of 100%.

The kinetic properties were evaluated by obtaining EIS of the sample before and after cycling up to 100 cycles at 100 mA g^−1^, The Nyquist plots are shown in Fig. S3(a)† and were fitted by a Randles circuit (Fig. S4(a) and (b)†), and the resultant fitting parameters are provided in Table 1. The estimated charge transfer resistance *R*_ct_ values for the WO_3_ electrode before and after 100 cycles were 109 Ω and 182 Ω, respectively. Using the following equation, we determined the diffusion coefficient of sodium ions:2
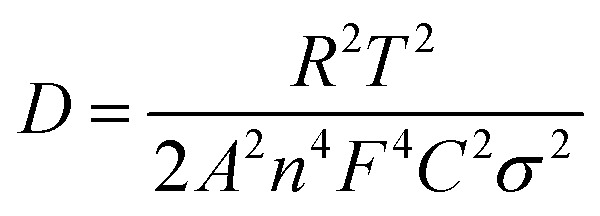
where *T*, *A* and *n* are the absolute temperatures, area of the electrode and the number of electrons during charge/discharge, respectively. *R* is the gas constant, *F* is the Faraday constant, *C* is the sodium ion concentration of sodium ions, and *σ* is the Warburg factor.

**Table 1:** The results from EIS and diffusion coefficients before and after cycling, obtained from the Nyquist plot

	*R*_1_ (Ω)	*R*_2_ (Ω)	*R*_3_ (Ω)	Slope (*σ*)	*D* (cm^2^ s^−1^)
Before cycling	6.15	109.55	—	4.78	5.01 × 10^−12^
After cycling	32	182	91	8.14	1.72 × 10^−12^

The Warburg factor is denoted by *σ* and the formula is *σ* = *Z*′/*ω*^−1/2^. It can be understood in terms of the impedance of the diffusion barrier layer and the semi-infinite diffusion impedance in the lower frequency slope that comes after the semicircle. The value of *σ* was found to be inversely proportional to the Na^+^ diffusion coefficient.^31^ The slope values (from Fig. S1(b)†) before and after 100 cycles were 4.78 and 8.14, respectively. The formula above could be used to determine the diffusion coefficients of Na ions, which were 5.01 × 10^−12^ and 1.72 × 10^−12^, respectively.

**Fig. 4:**
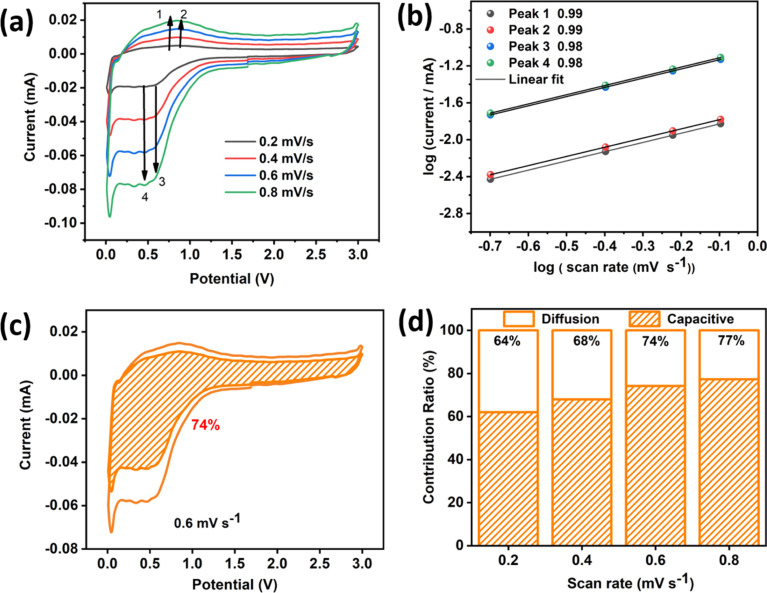
(a) CV plots at various scan rates ranging from 0.2 to 0.8 mV s^−1^. (b) Log(peak current, *i*) *vs.* log(scan rate, *v*), (c) the pseudocapacitive contribution (orange) at 0.6 mV s^−1^. (d) Storage contribution for various scan rates.

The reaction kinetics of WO_3_ was investigated *via* scanning cyclic voltammetry at different sweep rates ranging from 0.2–0.8 mV s^−1^, shown in Fig. 4(a). As the scan rate increased, these CVs showed the same contours and uninterrupted peak shifts. Therefore, the peak locations were similar to the CV results shown in Fig. 4(a) after the first cycle, indicating low polarization and strong electrochemical behavior. Eqn (3) and (4) were used to evaluate the charge storage contribution (diffusion-controlled contribution and capacitive contribution):^32^

3 *i* = *av*^*b*^

4 log *i* = *b* log *v* + log *a*

where *i* is the peak current and *v* is the scan rate, respectively; *a* and *b* are the changeable parameters.

By the log(*v*) *vs.* log(*i*) plot, the value of *b* can be calculated, which shows the capacitive and ion-diffusion contribution. In a capacitive-controlled process, *b* = 1.0, whereas in the ion diffusion process governing the electrochemical reaction, *b* = 0.5. When *b* falls within the range of 0.5 to 1.0, both capacitive and intercalation processes contribute to the storage of charge. For the as-synthesized WO_3_, the calculated *b* values were 0.99, 0.99, 0.98, and 0.98, for peaks 1, 2, 3, and 4, respectively as shown in Fig. 4(b). The charge storage contribution of WO_3_ can be further estimated by the following eqn (5):

5 *i* = *k*_1_*v* + *k*_2_*v*^1/2^

where, *k*_1_*v* corresponds to the capacitive contribution and *k*_2_*v*^1/2^ corresponds to the diffusive contribution. As illustrated in Fig. 4(c), the capacitive process of WO_3_ contributes 74% to the total capacity at the scan rate of 0.6 mV s^−1^. This result demonstrates that the material provides specific capacitance predominantly through a capacitive contribution. The variation of the capacitive and insertion contribution with respect to scan rates was also calculated and the changes are depicted in Fig. 4(d).

**Fig. 5:**
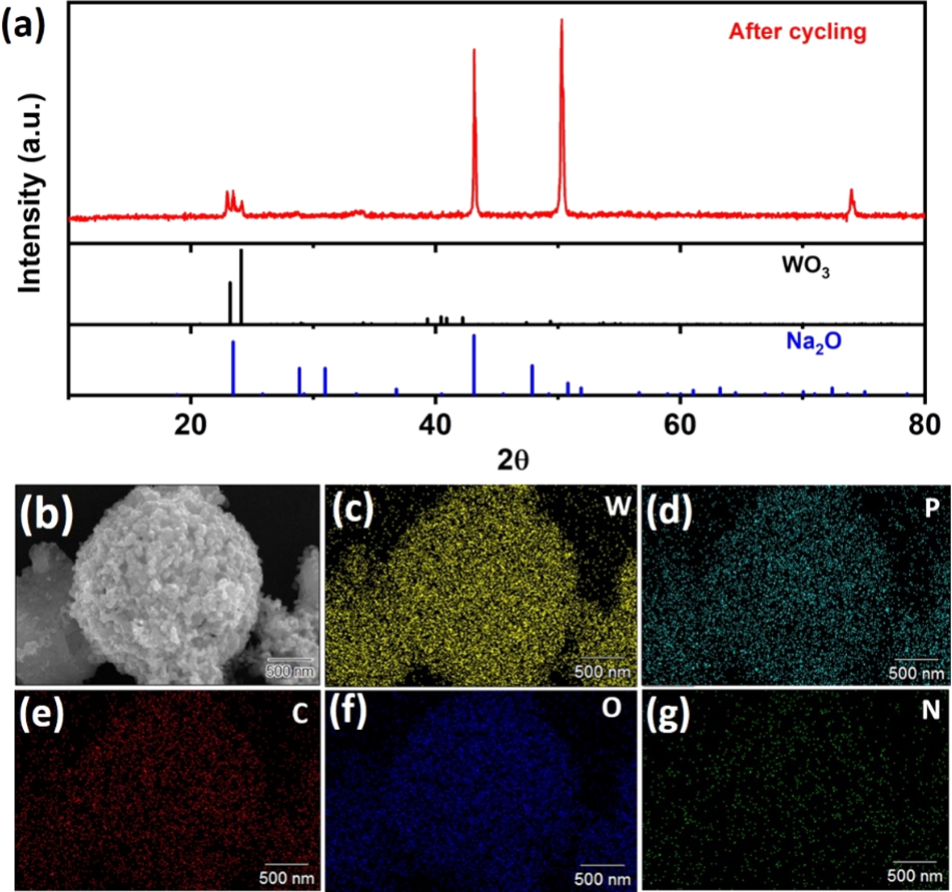
(a) XRD spectra of the electrode after cycling. (b) HRSEM image of the electrode material after cycling. (c) The corresponding elemental mapping images of W (tungsten), (d) P (phosphorus), (e) C (carbon), (f) O (oxygen), (g) N (nitrogen).

To investigate the phase change and structural changes that occur in the material upon charge–discharge cycling, the cycled cell was opened and the anode material was recovered. The cell was cycled up to 100 cycles at a current density of 100 mA g^−1^ before the sample was recovered. This post-electrochemical sample was analyzed using XRD and SEM. The XRD spectrum of the cycled anode material is shown in Fig. 5(a). The presence of tungsten trioxide and the absence of elemental tungsten in the XRD spectra indicates reversibility and the peaks for sodium oxide may have originated from the conversion reaction after the first cycle.

The SEM image and elemental mapping of the cycled sample are shown in Fig. 5(b–g). The SEM image did not reveal the nanosheets, which were likely obscured by PVDF, activated carbon, and dissociated electrolyte products on the surface. Despite this, the resilient spherical shape persisted, underscoring impressive structural stability. For further clarification of the microstructural changes in the post-cycling electrode material, two SEM images are provided in Fig. S5(a and b).† Elemental mapping indicates the presence of carbon, oxygen, phosphorus, and nitrogen, hinting at the accumulation of electrolyte, PVDF, and conductive carbon on the surface. The detailed observation of the transformation of the morphology and structure of the cycled anode proves that the sample's reversibility and structural stability are extremely good, enabling it to withstand continuous sodiation and desodiation.

## 3. Conclusion

We have successfully synthesized WO_3_ microspheres having a complex framework, made up of a nanosheet network. The as-synthesized anode material exhibited excellent electrochemical properties for Na-ion insertion. In response to ramping up current densities from 50 to 100, 150, 200, and 500 mA g^−1^, reversible capacities of 222.5, 124.0, 92.8, 70.0 and 40 mA h g^−1^ were achieved, respectively. Significantly, after the uninterrupted cycling with increasing current densities, a specific capacity as high as 220 mA h g^−1^ was regained at a current density of 50 mA g^−1^, claiming exquisite sodium storage reversibility. The reaction kinetics of WO_3_ was also investigated, and the capacitive process of WO_3_ contributed 74% to the entire capacity at the sweep rate of 0.6 mV s^−1^. Such a high value implies that the self-assembled WO_3_ microspheres offer ample sites for surface storage by the capacitive mechanism, which is reflected in rate capability, and enough space to buffer the volume change, which is correlated with the cycling stability.

## Conflicts of interest

The authors declare no potential conflicts of interest with respect to the research, authorship, and/or publication of this article.

## Supplementary Material

RA-014-D4RA01800A-s001
