# Correction: 3D-engineered WO_3_ microspheres assembled by 2D nanosheets with superior sodium storage capacity

**DOI:** 10.1039/d4ra90088j

**Published:** 2024-08-27

**Authors:** Shilpi Sengupta, C. Sudakar, Manab Kundu

**Affiliations:** a Electrochemical Energy Storage Laboratory, Department of Chemistry, SRM Institute of Science and Technology Chennai 603203 Tamil Nadu India manab.kundu@inl.int; b Multifunctional Materials Laboratory, Department of Physics, Indian Institute of Technology Madras Chennai 600036 India; c Nanomaterials for Energy Storage and Conversion, INL – International Iberian Nanotechnology Laboratory Av. Mte. José Veiga s/n 4715330 Braga Portugal

## Abstract

Correction for ‘3D-engineered WO_3_ microspheres assembled by 2D nanosheets with superior sodium storage capacity’ by Shilpi Sengupta *et al.*, *RSC Adv.*, 2024, **14**, 15706–15712, https://doi.org/10.1039/D4RA01800A

The authors regret that the one of the affiliations (affiliation *c*) was missing in the original manuscript. The corrected list of affiliations is as shown above.

The Royal Society of Chemistry apologises for these errors and any consequent inconvenience to authors and readers.

