# Supplementary material for: 3D-engineered WO3 microspheres assembled by 2D nanosheets with superior sodium storage capacity
Source: RSC Adv. 2024 May 14;14(22):15706–12. doi: 10.1039/d4ra01800a (PMC11091957; doi:10.1039/d4ra01800a)
Supplement: RA-014-D4RA01800A-s001 [file RA-014-D4RA01800A-s001.pdf]

Electronic Supplementary Material (ESI) for RSC Advances.
This journal is © The Royal Society of Chemistry 2024

Supporting information

3D- engineered WO_3 microspheres assembled by 2D nanosheets with superior sodium storage capacity

Shilpi Sengupta[1], C. Sudakar[2,] and Manab Kundu[1,] *

[1] Electrochemical Energy Storage Laboratory, Department of Chemistry, SRM Institute of Science and Technology, Chennai 603203, Tamil Nadu, India

2

[2] Multifunctional Materials Laboratory, Department of Physics, Indian Institute of Technology Madras, Chennai 600036, India

*Corresponding author: manabm@srmist.edu.in, csudakar@iitm.ac.in

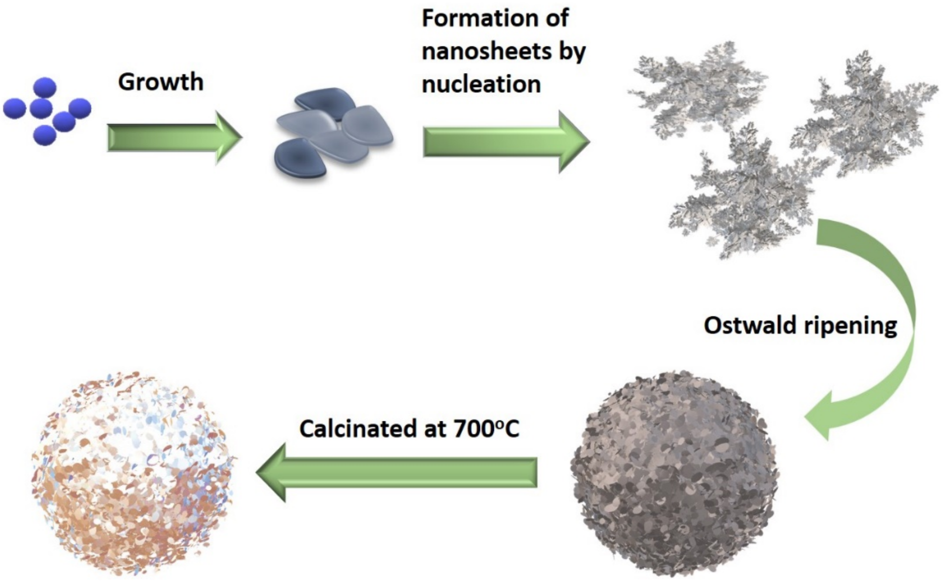

Figure S1. Schematic illustration of the WO_3 microspheres formation.

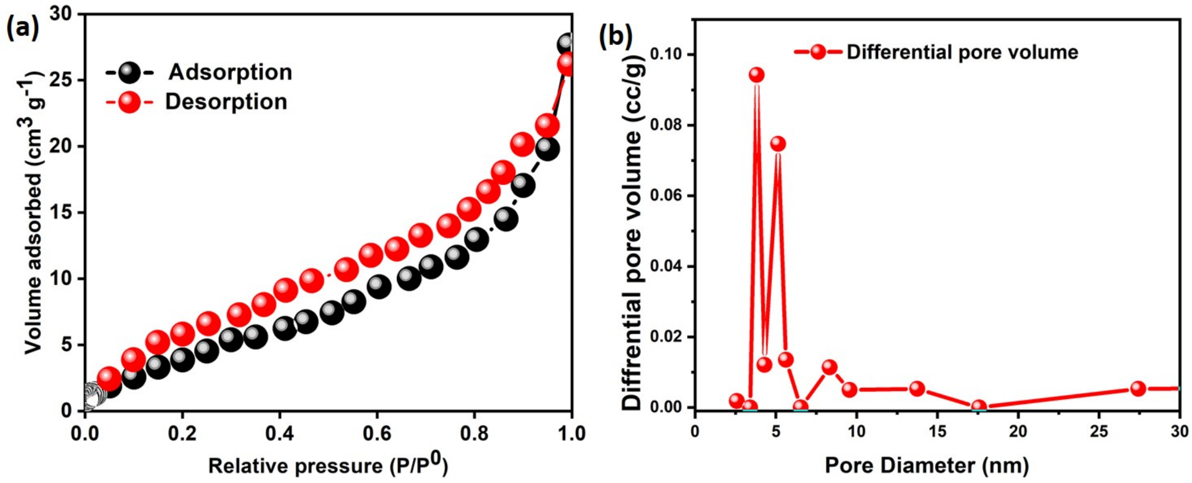

Figure S2. (a) Nitrogen adsorption-desorption isotherm curve of WO_3. (b) Pore size distribution plot of WO_3.

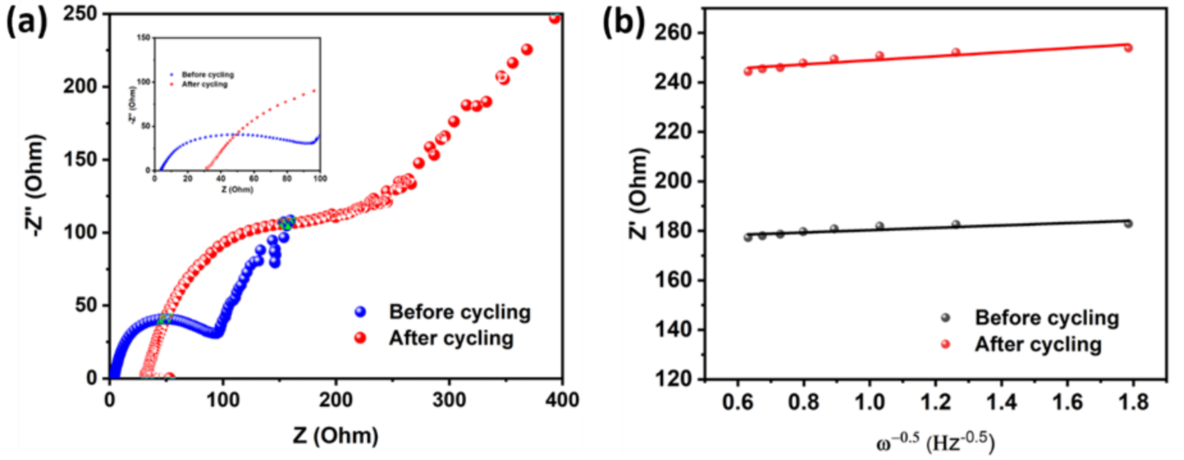

Figure S3. (a) Nyquist plot for WO3 before and after cycling. (b) Corresponding linear fitting results at low frequency

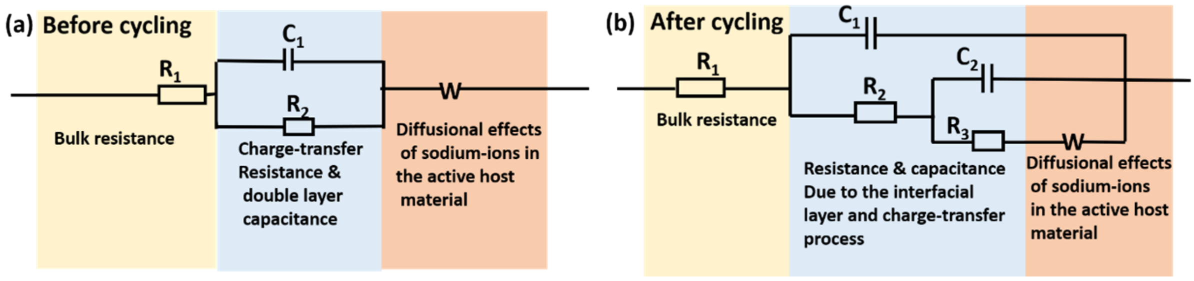

Figure S4. Equivalent circuit for fit the EIS (a) Before cycling and (b) After cycling.

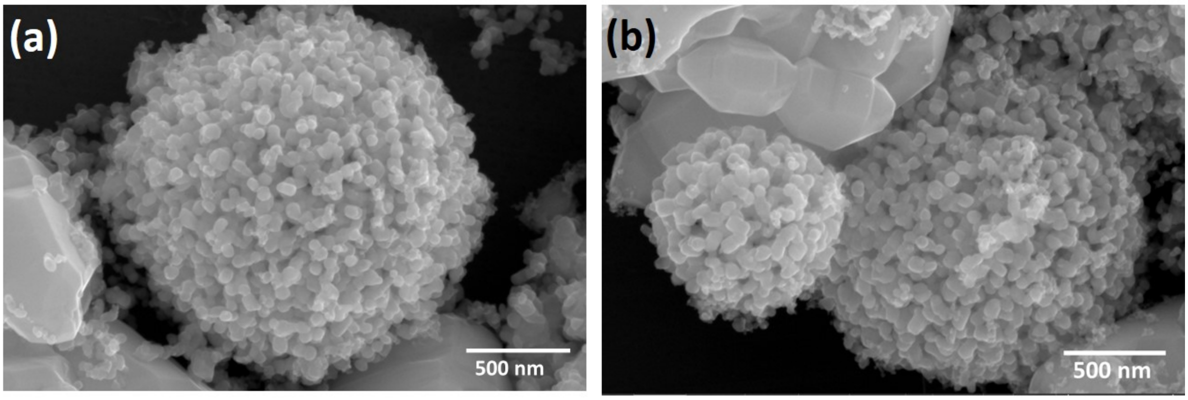

Figure S5. (a and b) HRSEM image of the electrode material after cycling.
